# Perceived family support and undernutrition among older outpatients of a Northern Nigerian hospital: A mixed methods study

**DOI:** 10.1371/journal.pone.0319626

**Published:** 2025-09-30

**Authors:** Abdulgafar Lekan Olawumi, Mohammed Abubakar Abiso, Zainab Abdulkadir, Aishatu Idris Umar, Lukman Abolaji Mohammed, Afisulahi Abiodun Maiyegun, Tiri Titilope Ogunyele, Hussaini Yusuf Magaji, Zainab Abdulazeez Umar, Abdullahi Ibrahim Haruna, Aliu Rasaki, Abdullahi Kabir Suleiman, Muazu Shuaibu Ishaqa, Ibrahim Danjummai Gezawa

**Affiliations:** 1 Geriatric Centre, Aminu Kano Teaching Hospital, Nigeria; 2 Department of Family Medicine, Aminu Kano Teaching Hospital, Kano, Nigeria; 3 Department of Family medicine, University of Maiduguri Teaching Hospital, Nigeria; 4 Usher Institute, College of Medicine and Veterinary Medicine, Deanery of Molecular, Genetic and Population Health Sciences, University of Edinburgh, United Kingdom; 5 Department of Family Medicine, Abubakar Tafawa Balewa Teaching Hospital, Bauchi, Nigeria; 6 Department of Family Medicine, Federal Medical Centre, Nguru, Nigeria; 7 Department of Family Medicine, Federal University Dutse,; 8 Department of Paediatrics, Gombe State University and Federal Teaching Hospital, Gombe, Nigeria; 9 Department of Family Medicine, Gombe State Specialist Hospital, Gombe, Nigeria; 10 Department of Internal Medicine, Aminu Kano Teaching Hospital/Bayero University Kano, Nigeria; Federal University of Agriculture Abeokuta, NIGERIA

## Abstract

**Background:**

Population ageing is increasing in developing countries like Nigeria, where declining death rates and high birth rates raise public health concerns, particularly regarding undernutrition. The shift toward nuclear family structures has diminished the support older adults receive from family members, compounded by economic challenges. This study seeks to explore the relationship between perceived family support and undernutrition among older adults, aiming to provide insights for enhancing health outcomes through improved family networks.

**Methods:**

The sequential mixed-methods study (cross-sectional study of 145 older adults followed by in-depth interviews of the identified undernourished older adults) was conducted in the Geriatric unit of a General Outpatient Clinic in Kano. Inferential statistical analyses were used to determine the associations between family support and undernutrition. Thematic analysis of the text data from the interviews was done using Nvivo® version 12 pro.

**Results:**

The mean age of respondents was 69.08 ± 7.82 (60–95) years; 76 (52.4%) were females. The prevalence of undernutrition was 15.2% and poor family support was 36.6%. Older age ≥ 75 (aOR=22.59, 95%CI = 5.45–93.57, *P* < 0.001), and poor family support (aOR=9.31, 95%CI = 2.32–37.42, *P* = 0.002) were the determinants of undernutrition in this study. Most undernourished older patients reported poor family interaction and satisfaction as the likely reasons for their condition.

**Conclusion:**

This study reported a high prevalence of undernutrition, with older age and poor family support as significant determinants. Undernutrition, driven by poor family support, financial hardships and limited food variety, emerged as recurring themes in the qualitative arm.

## Introduction

Population ageing was first observed in developed countries, but recent research shows similar trends emerging in developing nations [1]. Currently, Nigeria and other sub-Saharan African countries are in the second stage of demographic transition, characterized by declining death rates alongside persistently high birth rates. This transition leads to increased life expectancy and, as a result, a growing older population [2,3].

The rising number of older adults and their overall well-being is becoming a significant public health concern [3]. Ageing has a profound effect on their health, influencing wellness, illness, and disease states. Undernutrition is particularly common in this demographic and is one of the most critical factors negatively impacting their health [4]. It contributes to weakened immunity, heightened vulnerability to infections, prolonged hospital stays, and increased healthcare costs [5,6]. As a result, many studies on malnutrition in older adults tend to focus primarily on undernutrition [4–9].

Undernutrition can be categorized as either protein-energy wasting (traditionally referred to as kwashiorkor and marasmus) or due to specific nutrient deficiencies. In older adults, however, weight loss serves as the primary indicator of protein-energy malnutrition [5,7]. The understanding of undernutrition in the developed world is changing. Significant undernutrition impacts 5–10% of older individuals in nursing homes and can affect up to 50% of older patients upon discharge from the hospital [9].

Research has shown that weight loss in older adults doubles their risk of mortality, even among those who are overweight [10]. This heightened risk extends to individuals with obesity-related conditions such as diabetes. Furthermore, weight loss increases the likelihood of hip fractures and the need for institutional care in the older persons [11]. The six main causes of weight loss include anorexia, cachexia, malabsorption, hypermetabolism, dehydration, and sarcopenia [10,12]. Weight loss leads to elevated levels of circulating toxins, including insecticides stored in fat and triglycerides, which can result in the formation of highly atherogenic small dense low-density lipoprotein. This loss affects not only fat but also muscle and bone, increasing the risk of falls, hip fractures, and frailty [10]. Additionally, the reduction in fat can amplify the potential toxicity of fat-soluble medications, while a decrease in albumin can impact the efficacy and toxicity of protein-bound drugs [13].

In Nigeria, families play a vital role in supporting older citizens, particularly because pension and social security systems are inadequate, and retirement homes are almost nonexistent [14]. The extended family has traditionally served as a form of social insurance for the older adults, providing essential support during times of illness. This includes practical assistance such as meal preparation, medication management, and transportation to medical appointments, as well as emotional support [15]. However, the shift towards a nuclear family structure, driven by modernization, has significantly reduced the level of care and support that family members can provide. This decline is further exacerbated by economic hardships, unemployment, and rural-to-urban migration [16]. Given this context, it is crucial to examine and potentially harness the family structure and support systems to address issues such as undernutrition, and chronic diseases in older adults.

This mixed-methods study will provide a comprehensive understanding of the relationship between perceived family support and undernutrition, capturing both quantitative data on nutritional status and qualitative insights into the lived experiences of older adults. By exploring these dynamics, the research aims to determine the prevalence of undernutrition among the older adults and identify key factors that enhance or hinder nutritional support, ultimately informing interventions that leverage family networks to improve health outcomes. Furthermore, this study will not only contribute to the existing body of literature on geriatric health in Nigeria but also provide actionable insights for healthcare providers, policymakers, and families to better support the nutritional needs of older adults.

## Materials and methods

The sequential explanatory mixed-methods study (cross-sectional study followed by in-depth interviews of the identified undernourished older adults with poor family support) was conducted in the Geriatric unit of a General Outpatient Clinic in Kano. Kano is situated in the northwestern region of Nigeria and attracts individuals from diverse religious, ethnic, and occupational backgrounds. The hospital, which has 20 clinical departments and can accommodate over 800 inpatients, serves as a referral center for neighboring states and countries. As the primary care unit of the hospital, all older patients, except in emergencies, are assessed, treated, or referred to the appropriate sub-specialty units through the Geriatric unit of the General Outpatient Clinic (GOPC). According to the hospital’s medical records, the unit sees approximately 75 senior patients weekly.

The study population comprised older male and female patients aged 60 and above who visited the clinic during the 16-week study period, from March 12, 2024, to July 2, 2024. Older patients who provided written informed consent were recruited for the study. However, those with cognitive impairments, those who had lost body parts, or those needing emergency treatment, were excluded, as they might not cooperate with the study or could affect the accuracy of the anthropometric measurements.

### Sample size

Using Cochran’s formula: $n=Z^{\text{2}}p(\text{1}-p)/d^{\text{2}}$ and the following assumptions: [17] proportion of underweight (p) in older adults in Kano, Nigeria = 10.6%% [18], level of precision (d) = 5%, standard normal deviate (Z) for α error corresponding to 5% level of significance = 1.96, 90% response rate and correcting for finite study population, we arrived at a desired sample size of 145 participants.

### Sampling technique

For the quantitative arm, we used a systematic sampling technique to recruit participants. The sampling frame was 1200 (75x16). The first participant who fulfilled the criteria was selected using balloting. We then enrolled every subsequent 8^th^ (1200/145) patient who fulfilled the criteria until the desired sample size was reached. We recruited an average of 9 participants per week.

Purposive sampling was then used to select undernourished older patients with poor family support for in-depth interviews using the Key Informant Interview Guide.

### Data collection

#### Quantitative arm.

A trained research assistant administered the pretested, semi-structured, interviewer-administered questionnaire after obtaining written consent. The questionnaire includes information on the sociodemographic characteristics and medical history of participants. Participants’ height and weight were measured using a Seca Corporation® (Germany) stadiometer and weighing scale, with precision to the nearest 0.1 cm and 0.1 kg, respectively. For older patients with spinal curvatures or those in wheelchairs, height was estimated using half arm-span, measured from the midline at the sternal notch to the tip of the middle finger, with the total height calculated by doubling this measurement [19]. Body mass index [BMI = weight/height^2^] was calculated for the subjects and classified according to the WHO classification of obesity [20]. Undernutrition was defined as a BMI < 18.50 kg/m², while normal weight, overweight and obesity were categorized as BMI between 18.50–24.99 kg/m², BMI ≥ 25 kg/m² and BMI ≥ 30 kg/m² respectively.

We used the Perceived Social Support Family scale (PSS-Fa) to assess family support. This 20-item scale includes questions that respondents answer with “yes,” “no,” or “I don’t know.” Each “yes” response is scored as 1, while “no” and “I don’t know” receive a score of 0, allowing for a maximum score of 20. Scores of 11 and above indicate strong family support, scores between 7 and 10 signify weak support, and scores of 6 or lower indicate no family support [21]. Participants with no and weak family support were further combined to have “poor family support”. The PSS-Fa is a well-validated tool, demonstrating acceptable construct and factorial validity, as well as good internal consistency (Cronbach’s alpha of 0.85) in studies involving Ghanaian patients [22].

#### Qualitative arm.

In-depth interviews were conducted for undernourished older patients with poor family support who were identified at the end of the quantitative study, using the Key Informant Interview Guide. Each session lasted for 30 to –45 minutes. It was audio-recorded with a moderator and an assistant in attendance who were both trained in the conduct of in-depth interviews and experts in qualitative studies. The assistant recorded the discussion as well as observed and documented the process for the moderator. Participants were asked open-ended questions on their views about their nutritional status in relation to lack of family support, and their perceptions on their family structure, support and interaction.

### Statistical analysis

We coded, organized, and analyzed the data using SPSS software version 22 (IBM Corp., Armonk, NY, USA). After cleaning the data, continuous variables were summarized using means with standard deviations (SD) or medians with interquartile ranges (IQR). Categorical variables were presented as frequencies and percentages. For bivariate analysis, Pearson’s Chi-square test was employed to compare frequencies, while Fisher’s exact test was utilized when the assumptions of the Chi-square test were not met. Variables with a p-value ≤ 0.05 at the bivariate level, along with those deemed contextually relevant, were included in a binary logistic regression model. A Type I error rate of 5% was set for all hypothesis tests.

Thematic analysis of the text data from the key informant interviews (KII) was conducted using NVivo® version 12 Pro, a qualitative software designed for data storage, coding, and theme development. NVivo was utilized for transcribing the recorded interviews, coding the responses into themes, identifying core themes, conducting systematic team-based coding, creating a Numeric Content Analysis (NCA) table, and preparing the analyzed data for publication. Theme development and revision occurred iteratively, with themes emerging from the data. To enhance the credibility of the findings, member checking was employed, and the results were summarized with relevant verbatim quotes included as necessary.

### Ethical considerations

Ethical approval was obtained from the Aminu Kano Teaching Hospital Research Ethics Committee (NHREC/21/08/2008/AKTH/EC/2092). A written informed consent was gotten from each participant before the commencement of the study. Participants who were diagnosed with undernutrition were managed and referred appropriately.

## Results

A total of 145 respondents (100% response rate) participated in the study. Their ages ranged from 60 to 95 years, with a mean age of 69.08 years (SD ± 7.82). As illustrated in Table 1, the majority (71.0%) of respondents were under 75 years of age, while 29.0% fell into the age category of 75 years and older. Most participants were female (52.4%), of the Hausa ethnic group (64.8%), and identified as Muslim (95.9%). A significant number lived in extended family settings (86.2%), had no formal education (73.1%), and earned less than ₦70,000 (approximately 43 USD) per month (89.0%). About two-third of the respondents (63.4%) reported having strong family support, while 30.4% had weak support and 6.2% reported no family support. Consequently, the prevalence of poor family support in the study was 36.6% Table 1.

**Table 1:** Factors associated with Undernutrition among older adults (n = 145).

Variables	Total (n = 145)	Undernutrition (n = 22)	No undernutrition (n = 123)	χ^2^	P value
**Age groups (Years)**				35.210	< **0.001**
< 75	103(71.0%)	4(18.2%)	99(80.5%)		
≥ 75	42(29.0%)	18(81.8%)	24(19.5%)		
*Mean ± SD*	***69.08 ± 7.82***				
**Sex**				9.164	**0.002**
Male	69(47.6%)	17(77.3%)	52(42.3%)		
Female	76(52.4%)	5(22.7%)	71(57.7%)		
**Marital status**				3.801*	0.104
Married	83(57.2%)	17(77.3%)	66(53.7%)		
Divorce/separated	5(3.4%)	0(0.0%)	5(4.1%)		
Widow	57(39.4%)	5(22.7%)	52(42.2%)		
**Religion**				1.119	0.290
Islam	139(95.9%)	22(100.0%)	117(95.1%)		
Christianity	6(4.1%)	0(0.0%)	6(4.9%)		
**Tribe**				1.708*	0.838
Hausa	94(64.8%)	13(59.1%)	81(65.9%)		
Fulani	36(24.8)	7(31.8%)	29(23.6%)		
Yoruba	3(2.1)	0(0.0%)	3(2.4%)		
Igbo	1(0.7%)	0(0.0%)	1(0.8%)		
Others	11(7.6%)	2(9.1%)	9(7.3%)		
**Educational level**				2.299*	0.588
No	106(73.1%)	15(68.3%)	91(74.0%)		
Primary	13(8.9%)	1(4.5%)	12(9.8%)		
Secondary	4(2.8%)	1(4.5%)	3(2.4%)		
Tertiary	22(15.2%)	5(22.7%)	17(13.8%)		
**Occupation**				8.396	**0.015**
Employed	59(40.7%)	11(50.0%)	48(39.0%)		
Unemployed	68(46.9%)	5(22.7%)	63(51.2%)		
Retired	18(12.4%)	6(27.3%)	12(9.8%)		
**Family structure**				0.482	0.487
Nuclear	20(13.8%)	2(9.1%)	18(14.6%)		
Extended	125(86.2%)	20(90.9%)	105(85.4%)		
**Family support**				18.544	**< 0.001**
Poor	53(36.6%)	17(77.3%)	36(67.9%)		
Strong	92(63.4%)	5(22.7%)	87(94.6%)		
**Monthly income (USD)**				0.100*	0.752
< 43	129(89.0%)	20(90.9%)	109(88.6%)		
≥ 43	16(11.0%)	2(9.1%)	10(11.4%)		
*Median (IQR)*	***12.1 (6.2–24.4)***				
**Residence**				4.822	**0.028**
Rural	64(44.1%)	5(22.7%)	59(48.0%)		
Urban	81(55.9%)	17(77.3%)	64(52.0%)		

*Others: Kanuri, Ebira, Edo, Igala, Tiv. 1 USD = *₦*1650*. ***Bold:*** Statistically significant. * Fisher’s Exact Test.

**Fig 1:**
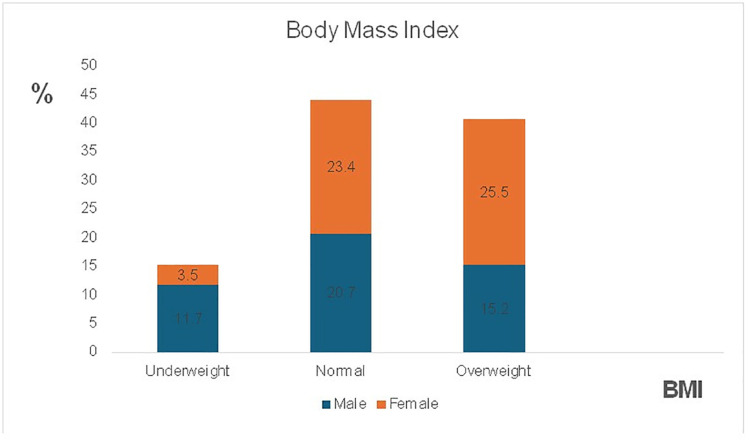
BMI pattern of respondents by gender (p = 0,006).

Fig 1 presents the distribution of body mass index (BMI) among the respondents, showing that 15.2% were underweight, 44.1% had a normal BMI, 40.7% were classified as overweight. Thus, the prevalence of undernutrition in this study is 15.2% (95% CI: 9.3–21.0).

Age (χ^2^ = 32.210, p < 0.001), sex (χ^2^ = 9.164, p = 0.002), occupation (χ^2^ = 8.396, p = 0.015), residence (χ^2^ = 4.822, p = 0.028), and family support (χ^2^ = 18.544, p < 0.001) are significantly associated with undernutrition among older adults in this study. Respondents within the age group of ≥ 75 years (81.8%), male (77.3%), employed (50.0%), urban residents (77.3%) and with poor family support (77.3%) have the highest percentage of undernutrition. While not statistically significant, undernutrition was more prevalent among those living in extended family settings (90.9%) than those in the nuclear settings (9.1%). Notably, only 2 (9.1%) of the respondents who earned above 43 USD were found to be undernourished Table 1.

Logistic regression of the associated factors with undernutrition in Table 2 revealed that older age group of 75 years and above (aOR=22.59, 95%CI = 5.45–93.57, *P* < 0.001), and poor family support (aOR=9.31, 95%CI = 2.32–37.42, *P* = 0.002), were the significant determinants of undernutrition among the older adults in this study.

**Table 2:** Binary logistic regression analysis of factors associated with undernutrition among older adults.

Variables	Adjusted Odd ratio	Confidence interval	*p*-value
**Age groups (Years)**			
< 75	Reference		
≥ 75	22.59	5.45–93.57	**< 0.001**
**Sex**			
Male	1.17	0.20–6.90	0.865
Female	Reference		
**Occupational**			
Employed	0.40	0.08–1.95	0.255
Unemployed	0.10	0.01–1.14	0.064
Retired	Reference		
**Family support**			
Poor	9.31	2.32–37.42	**0.002**
Strong	Reference		
**Residence**			
Rural	Reference		
Urban	1.45	0.25–8.43	0.681

**Bold:** Statistically significant association with undernutrition at *P* ≤ 5%.

### In-depth Interviews (IDI)

In-depth qualitative interviews were conducted with only 10 out of the 17 undernourished older patients with poor family support due to reaching information saturation.

### Demography, family structure and dynamics

The respondents, aged 60 to 83 years, comprised both males and females with varied marital statuses (widowed and married). Most had little to no formal education but possessed some Islamic education. Their occupations included farming, petty trading, and homemaking. Majority live with family members (children, grandchildren, or spouses). In some cases, they live with extended family members like in-laws. Family structures are varied, with the majority being extended households. In addition, the interviewed participants were found to be generally older, widowed, less educated, and economically disadvantaged compared to the other participants.

### Family support system and communication

Family members (children, spouses, or grandchildren) provide financial and emotional support.

Support systems are stronger in households with multiple caregivers. Some families face economic challenges, limiting their ability to provide adequate support. A 64-year-old male retiree said:

*“My children do their best to provide me with the necessary support, but economic challenges have constrained their efforts. Most of them are civil servants with limited income.”*

Communication is generally good; most older respondents can reach out to family members when needed. However, a few respondents reported limited interaction or dependency on initiating contact with their family members. A 69-year-old female, widowed for 21 years said: *“My children have settled in different states, and I rely solely on phone calls to communicate with them, which often happen weeks or even months apart due to their busy schedules. I currently live with my grandchild, who is 8 years old.”*

### Nutritional support, food preparation and access

Farming and small businesses are major sources of income, but they are often insufficient. Many seniors depend entirely on family members for financial and nutritional support. Economic hardships within families limit their ability to provide diverse and nutritious meals.

Their staple foods include “*tuwo and mia kuka*” (made from maize flower), “*gurasa*” (local bread), “Kunu” (made from maize or millet), spaghetti, rice, beans and “*dan wake*” (made from wheat flower). Fruits and vegetables, meat, fish or egg are taken on few occasions when brought by their children. Meals are generally prepared by family members (wives, daughters, granddaughters). Some respondents cook for themselves if no one else is available. Delays in meal preparation and limited food variety are reported by some respondents. An 80-year-old female who had only Islamic education said:

*“I live with my daughter, son-in-law, and granddaughter, who usually handles cooking and food preparation. However, meals are often delayed, so I sometimes have to prepare my own food. The delays are mostly due to high family commitments and occasional food unavailability.”*

### Impacts of poor family support on nutritional needs

Nutritional care varies widely. Few receive a balanced diet (meat, fish, fruits), while most rely on basic foods due to financial constraints. Inadequate nutrition is linked to malnutrition and associated health issues. Dependence on family for transport to healthcare facilities is common.

A 64-year-old male retiree said: *“I used to face challenges in maintaining consistent and balanced meals. My feeding pattern often depends on the availability of affordable options, which may lack essential nutrients. Additionally, the lack of support in meal planning or preparation further contributes to my irregular eating habits.”*

An 83-year-old male farmer with Islamic education said: *“Even though we farm, we can’t afford to buy different kinds of food besides what we grow. I don’t have much appetite for food, and I don’t get regular support or help with food.”*

An 82-year-old female stay-at-home individual with no formal education said: *“We depend on staple food supplies that are not always balanced. My children help with food sometimes, but we don’t always get a variety of nutritious meals. It’s also hard to get or make different kinds of food because I live with my 13-year-old granddaughter, who doesn’t know how to cook many dishes.”*

## Discussions

Nutritional problems among the increasing population of older adults, either due to the process of ageing or socio-economic factors, is a global cause for concern. This sequential expalanatory mixed-methods study was conducted to assess the association between perceived family support and undernutrition, and also explore other socio-economic factors that influence the nutritional status of the older adults.

### Quantitative outcomes

The prevalence of undernutrition in this study is 15.2%. This is high and aligns with the findings from Kamrup District, India (15%),^5^ Harar, Eastern Ethiopia (16.6%) [23], Sodo Zuriya District, Ethiopia (17.1%) [24], South-Central Nepal (19.8%) [25]. The rate observed in our study was lower than those reported in other studies, such as Lagos, southwestern Nigeria (35.7%) [26], Kano, northwestern Nigeria (25.9%) [27], Gondar Town, Northwest Ethiopia (21.9%) [28], and Nepal (24.8%) [29]. Conversely, it was higher than the prevalence reported in Ibadan, southwestern Nigeria (7.8%) [4], Kano, northwestern Nigeria (10.6%) [18], Pakistan (5.53%) [30], and Malaysia (7.3%) [31]. These differences may stem from variations in study populations, as our study focused on older outpatients, while others included older adults living in the community or nursing homes. Furthermore, disparities in socioeconomic factors, health conditions, and medication use likely contribute to the observed variations.

Amongst the various socio-demographic characteristics analysed, advancing age, sex, occupation, residence, and family support were associated with undernutrition on bivariate analysis. But on further analysis with logistic regression, only advancing age and poor family support were the independent determinants of undernutrition among older adults in this study. Respondents aged 75 years and above, males, retirees, urban residents, and those with poor family support showed the highest rates of undernutrition. Although not statistically significant, undernutrition was more common among individuals living in extended family settings compared to those in nuclear families. None of the respondents earning above 86 USD, though few, were found to be undernourished.

The strong association between advancing age and undernutrition identified in this study (aOR 22.59, CI 5.45–93.57, p < 0.001) aligns with findings from several other studies [18,23–27]. This relationship may be attributed to the physiological and pathological changes that accompany aging, which can lead to the development of comorbid conditions, functional impairments, prolonged medication use, feeding difficulties, and ultimately, undernutrition.^5^ The significant association between undernutrition and poor family support is consistent with other studies [2,17–19].

This study revealed a significant relationship between poor perceived family support and the undernutrition (aOR 9.31, CI 2.32–37.42, p = 0.002), such that older patients with poor perceived family support are nine times more like to be undernourished than those with strong perceived family support. This is because family with effective and adequate support will always share time with their older members including eating and sharing feelings together [32]. Although there were few literatures to compare with, similar findings were reported by Fattah *et al* in Libya and Damayanthi *et al* in Sri Lanka [32,33]. In addition, similar findings was reported by Wen et al. among older Hispanic Americans with type 2 diabetes, which revealed an inverse relationship between perceived family support and barriers to diet and nutrition [34]. Beyond the multifactorial impact of diabetes on diet and nutrition, the Hispanic population is often characterized by certain family structure dynamics such as endogamy, delayed marriage, and high divorce rates. These dynamics can weaken family cohesion and intergenerational support systems, particularly for older family members. This may result in less consistent financial and emotional assistance, reducing their ability to access or prioritize nutritious food and ultimately impacting their overall nutrition [35].

### Qualitative outcomes

#### Family support system and communication.

The findings highlight the critical role of family support systems in ensuring the well-being of older adults. Financial and emotional support from children, spouses, or grandchildren is a cornerstone of their care. However, economic challenges, particularly in families with limited income, often constrain the level of support they can provide. This was evident from the accounts of respondents like the 64-year-old retiree, who acknowledged his children’s efforts but noted their struggles due to low-paying civil service jobs. A study by Aboderin in 2004 on intergenerational support in sub-Saharan Africa also described how economic pressures on younger family members affect their ability to care for aging relatives [36]. Communication within families was generally positive, with most respondents able to reach out to their family members when needed. However, some respondents reported limited interaction, especially when family members lived far away or were preoccupied with their busy schedules. Donovan *et al* in 2020, also found that older adults with children living far away were more likely to experience social isolation, relying heavily on occasional phone calls or virtual communication for emotional support [37].

### Nutritional support, food preparation, and access

The reliance on staple foods with occasional inclusion of fruits, vegetables, or protein-rich foods like meat and fish, emphasized the limited dietary variety available to many respondents. Meal preparation was predominantly managed by family members. However, high family commitments and food unavailability often lead to delays, forcing some respondents to prepare their own meals despite physical or cognitive challenges. This is consistent to the report of Rusu *et al* where older adults often resort to preparing their own meals when family members are unavailable, despite physical or cognitive limitations, which increases their risk of inadequate nutrition and related health complications [38].

### Impacts of poor family support on nutritional needs

Financial hardships and limited food variety as drivers of undernutrition, emerged as recurring themes in this study. Respondents frequently relied on affordable, basic foods that lacked essential nutrients, further exacerbating health risks. Dependence on family members extends beyond food provision to emotional and economic support. For example, the 64-year-old retiree’s irregular eating habits and limited access to nutrient-rich foods reflect the broader challenge of addressing nutritional needs in economically disadvantaged households. Similarly, the 83-year-old farmer’s reliance on self-grown crops and the 82-year-old stay-at-home woman’s struggle with food preparation due to her young granddaughter’s inexperience highlight the vulnerability of older adults living in resource-limited settings.

### Limitations and recommendations

The limitations of this study include the lack of biochemical and hematological assessments of undernutrition, and potential temporal bias for recall questions in both quantitative and qualitative arms. Additionally, as a cross-sectional study, causality between perceived family support and undernutrition cannot be established, necessitating longitudinal research.

In our logistic regression analysis, the identified predictors of undernutrition (advancing age and poor family support) were associated with wide confidence intervals. This reflects imprecision in the estimates, which is likely due to the relatively small sample size and sparse data for some predictor categories. Such situations are known to reduce the stability of logistic regression estimates and inflate confidence intervals [39]. While the direction of association remained consistent with existing literature, the width of the intervals suggests that these findings should be interpreted with caution. Future studies with larger sample sizes and more balanced data distributions are needed to provide more precise estimates.

Another limitation of the study is that clinical data that could explain malnutrition, such as functional status or cognition, were not assessed. Despite these limitations, the findings provide valuable evidence to advocate for measures aimed at strengthening family support systems, ensuring access to diverse and nutritious foods, empowering older adults financially through pensions or social programs, and promoting community-based nutritional interventions.

## Conclusion

Although, comparable with previous studies, the prevalence of undernutrition among older persons was high in this study. Older age and poor family support were the significant determinants of undernutrition. In the qualitative arm, undernutrition influenced by poor family support, financial constraints and a lack of dietary diversity, emerged as a consistent theme in this study. These findings emphasize the interconnected challenges of economic constraints, family dynamics, and nutritional deficiencies in the lives of older adults. Strengthening family support through health education and counselling to enhance awareness, active family involvement in care, and community-based programs that reduce isolation will resolve these challenges. Complementary measures such as improved access to nutrition, financial empowerment, and social protection schemes will further enhance families’ capacity to care for older adults.
